# Supplementary material for: Excess Iodine Consumption Induces Oxidative Stress and Pancreatic Damage Independently of Chemical Form in Male Wistar Rats: Participation of PPAR-γ and C/EBP-β
Source: Biology (Basel). 2024 Jun 25;13(7):466. doi: 10.3390/biology13070466 (PMC11273434; doi:10.3390/biology13070466)
Supplement: Supplementary file 1 [file biology-13-00466-s001.zip › biology-3056824-supplementary.pdf]

Supplementary material

File S1: details of western blot membranes

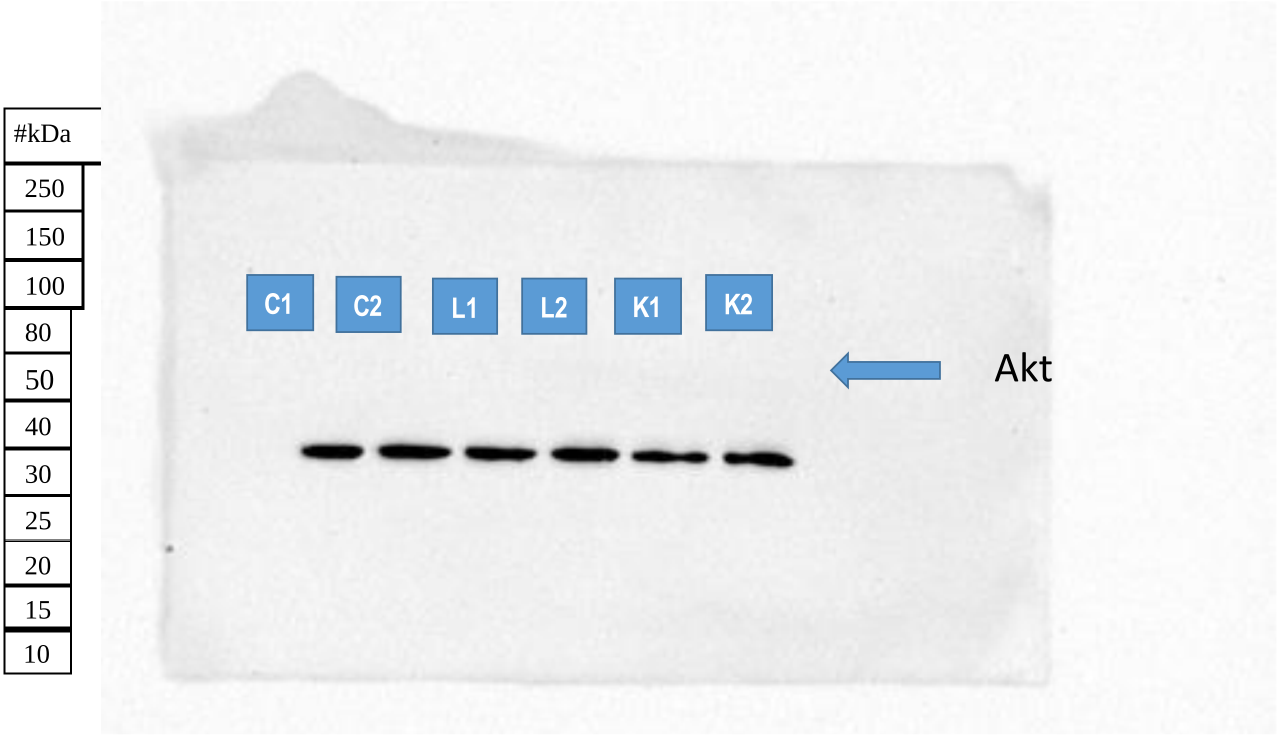

Figure S1. Western Blot membrane of Akt protein (56 kDa) detected with anti-Akt (1:1500, Cell Signaling C67E7). SDS page were transferred to nitrocellulose membranes (0.2 µm pore size; Bio-Rad) by semidry electroblotting (100 V, 2 hours). Membranes were incubated with an anti-rabbit secondary antibody (Cell Signaling 65-6120; 1:4000) and detection was performed using an enhanced chemiluminescence protocol (SuperSignal West Pico PLUS Chemiluminescent Substrate [ThermoFisher]). #Weight marker (kDa) used: Protein Ladder BioLabs p7703s, 10 to 250 kDa. Blot images, were converted to grayscale with ImageJ software (v. 1.52a, National Institute of Health, USA). C1-2: control group; L1-2: Lugol group; K1-2, KIO3 group.

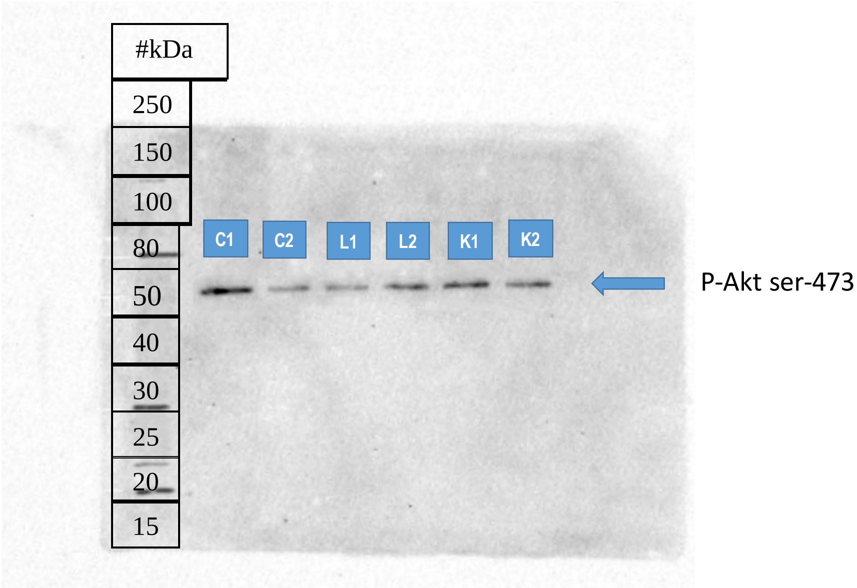

Figure S2. Western Blot membrane of Akt ser473 protein (56 kDa) detected with anti-fosfo-Akt (1:1000, Cell Signaling 9271). SDS page were transferred to nitrocellulose membranes (0.2 µm pore size; Bio-Rad) by semidry electroblotting (100 V, 2 hours). Membranes were incubated with an anti-rabbit secondary antibody (Cell Signaling 65-6120; 1:4000) and detection was performed using an enhanced chemiluminescence protocol (SuperSignal West Pico PLUS Chemiluminescent Substrate [ThermoFisher]). #Weight marker (kDa) used: Protein Ladder BioLabs p7703s, 10 to 250 kDa. Blot images, were converted to grayscale with ImageJ software (v. 1.52a, National Institute of Health, USA). C1-2: control group; L1-2: Lugol group; K1-2, KIO3 group.

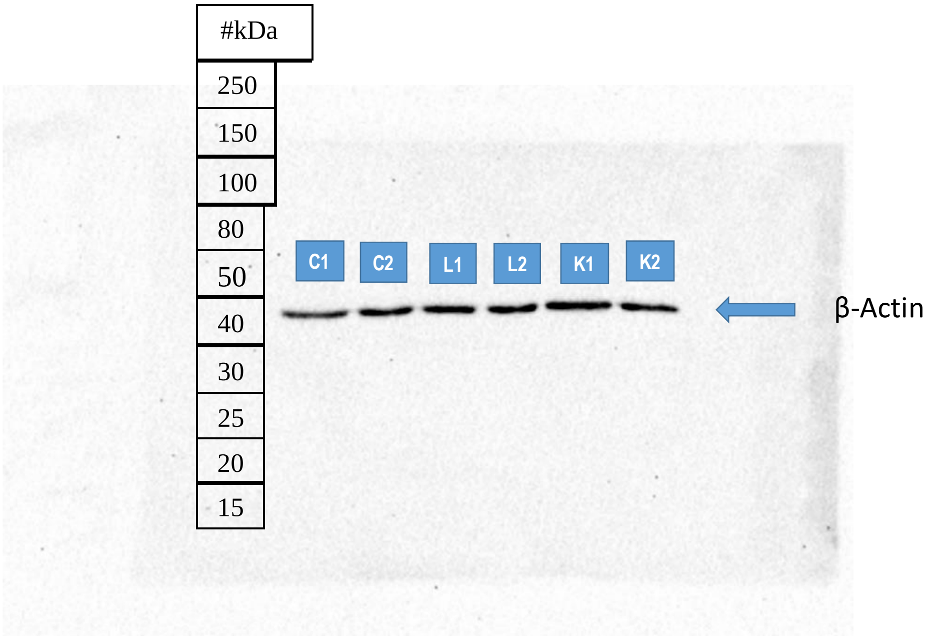

Figure S3. Western Blot membrane of beta-actin (43 kDa) detected with anti-fosfo-actin (1:1000, Cell Signaling). SDS page were transferred to nitrocellulose membranes (0.2 µm pore size; Bio-Rad) by semidry electroblotting (100 V, 2 hours). Membranes were incubated with an anti-rabbit secondary antibody (Cell Signaling 65-6120; 1:4000) and detection was performed using an enhanced chemiluminescence protocol (SuperSignal West Pico PLUS Chemiluminescent Substrate [ThermoFisher]). #Weight marker (kDa) used: Protein Ladder BioLabs p7703s, 10 to 250 kDa. Blot images, were converted to grayscale with ImageJ software (v. 1.52a, National Institute of Health, USA). C1-2: control group; L1-2: Lugol group; K1-2, KIO3 group.

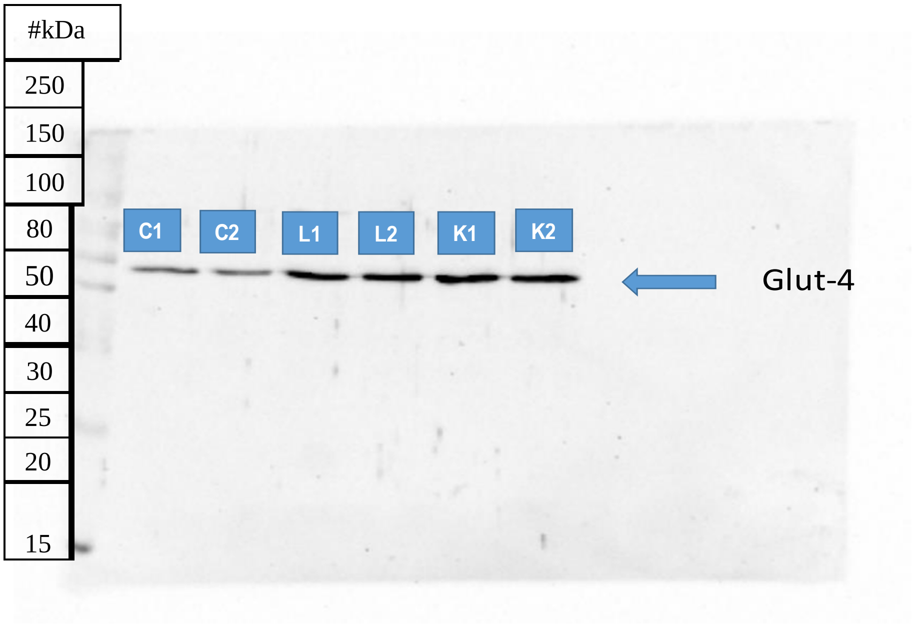

Figure S4. Western Blot membrane of GLUT4 protein (55 kDa) detected with anti-GLUT4 (1:2000, ab654 Abcam). SDS page were transferred to nitrocellulose membranes (0.2 µm pore size; Bio-Rad) by semidry electroblotting (100 V, 2 hours). Membranes were incubated with an anti-rabbit secondary antibody (Cell Signaling 65-6120; 1:4000) and detection was performed using an enhanced chemiluminescence protocol (SuperSignal West Pico PLUS Chemiluminescent Substrate [ThermoFisher]). #Weight marker (kDa) used: Protein Ladder BioLabs p7703s, 10 to 250 kDa. Blot images, were converted to grayscale with ImageJ software (v. 1.52a, National Institute of Health, USA). C1-2: control group; L1-2: Lugol group; K1-2, KIO3 group.

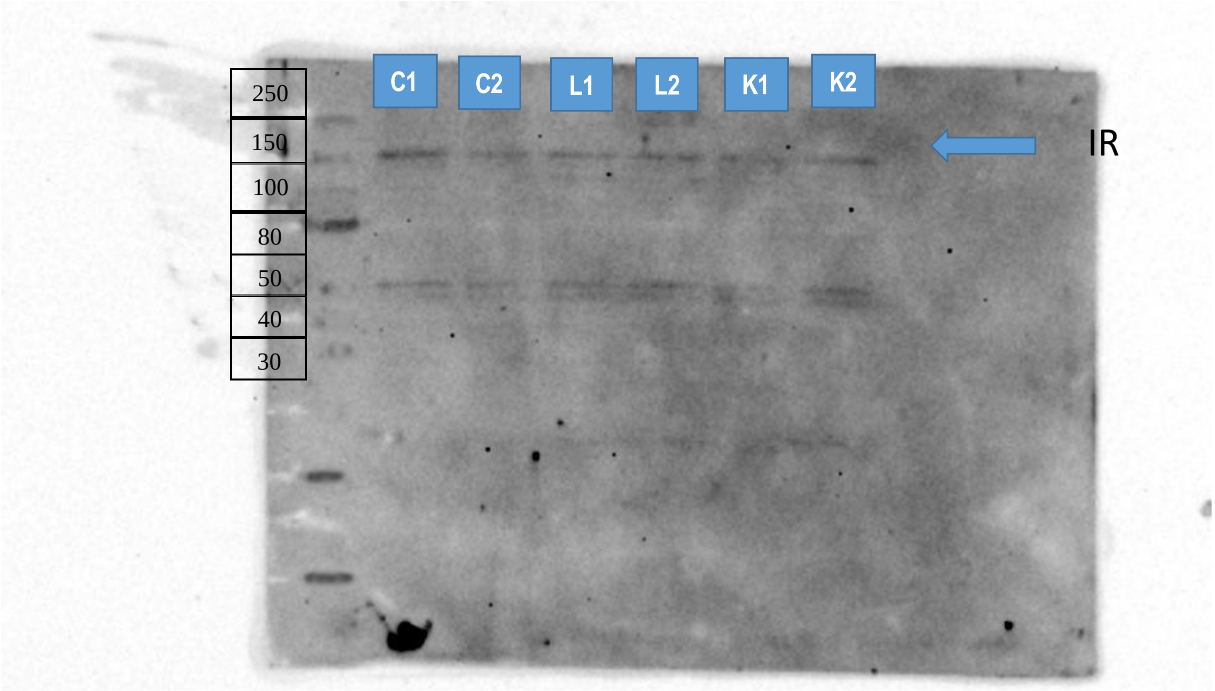

Figure S5. Western Blot membrane of insulin receptor protein (125 kDa) detected with anti-Insulin receptor alpha ((1:2000, ab5500 Abcam). SDS page were transferred to nitrocellulose membranes (0.2 µm pore size; Bio-Rad) by semidry electroblotting (100 V, 2 hours). Membranes were incubated with an anti-rabbit secondary antibody (Cell Signaling 65-6120; 1:4000) and detection was performed using an enhanced chemiluminescence protocol (SuperSignal West Pico PLUS Chemiluminescent Substrate [ThermoFisher]). #Weight marker (kDa) used: Protein Ladder BioLabs p7703s, 10 to 250 kDa. Blot images, were converted to grayscale with ImageJ software(v. 1.52a, National Institute of Health, USA). C1-2: control group; L1-2: Lugol group; K1-2, KIO3 group.
